# Engineering a thermostable *Halothermothrix orenii β*-glucosidase for improved galacto-oligosaccharide synthesis

**DOI:** 10.1007/s00253-015-7118-8

**Published:** 2015-12-01

**Authors:** Noor Hassan, Barbara Geiger, Rosaria Gandini, Bharat K. C. Patel, Roman Kittl, Dietmar Haltrich, Thu-Ha Nguyen, Christina Divne, Tien Chye Tan

**Affiliations:** AlbaNova University Center, School of Biotechnology, KTH Royal Institute of Technology, Roslagstullsbacken 21, S-10691 Stockholm, Sweden; Food Biotechnology Laboratory, BOKU-University of Natural Resources and Life Sciences Vienna, 1190 Vienna, Austria; Microbial Gene Research and Resources Facility, School of Biomolecular and Physical Sciences, Griffith University, Brisbane, QLD 4111 Australia; Department of Medical Biochemistry and Biophysics, Karolinska Institute, Scheelelaboratoriet, Scheeles väg 2, S-17177 Stockholm, Sweden

**Keywords:** *β*-Glucosidase, *β*-Galactosidase, Halothermophile, *Halothermothrix*, Lactose conversion, Galacto-oligosaccharides, Transglycosylation mutants

## Abstract

Lactose is produced in large amounts as a by-product from the dairy industry. This inexpensive disaccharide can be converted to more useful value-added products such as galacto-oligosaccharides (GOSs) by transgalactosylation reactions with retaining *β*-galactosidases (BGALs) being normally used for this purpose. Hydrolysis is always competing with the transglycosylation reaction, and hence, the yields of GOSs can be too low for industrial use. We have reported that a *β*-glucosidase from *Halothermothrix orenii* (*Ho*BGLA) shows promising characteristics for lactose conversion and GOS synthesis. Here, we engineered *Ho*BGLA to investigate the possibility to further improve lactose conversion and GOS production. Five variants that targeted the glycone (−1) and aglycone (+1) subsites (N222F, N294T, F417S, F417Y, and Y296F) were designed and expressed. All variants show significantly impaired catalytic activity with cellobiose and lactose as substrates. Particularly, F417S is hydrolytically crippled with cellobiose as substrate with a 1000-fold decrease in apparent *k*_cat_, but to a lesser extent affected when catalyzing hydrolysis of lactose (47-fold lower *k*_cat_). This large selective effect on cellobiose hydrolysis is manifested as a change in substrate selectivity from cellobiose to lactose. The least affected variant is F417Y, which retains the capacity to hydrolyze both cellobiose and lactose with the same relative substrate selectivity as the wild type, but with ~10-fold lower turnover numbers. Thin-layer chromatography results show that this effect is accompanied by synthesis of a particular GOS product in higher yields by Y296F and F417S compared with the other variants, whereas the variant F417Y produces a higher yield of total GOSs.

## Introduction

About 150–200 million tons of lactose are generated each year from liquid whey (Smithers, 2008). Lactose can be conveniently hydrolyzed into glucose and galactose by the use of *β*-galactosidases (BGALs). In cases where the enzyme displays significant transglycosylation activity, hydrolysis can be combined with the transfer of hydrolysis products onto suitable acceptor molecules to form new value-added compounds such as galacto-oligosaccharides (GOSs).

The main producers of GOSs are BGALs produced by various microorganisms such as *Aspergillus oryzae*, *Aspergillus niger*, *Kluyveromyces lactis*, and *Kluyveromyces fragilis* (Torres et al. 2010; Gosling et al. 2010). A number of commercial and non-commercial BGALs from different sources have been evaluated for their ability and efficiency to produce GOSs (e.g*.*, Petzelbauer et al. 2000; Jørgensen et al. 2001; Hung et al. 2002; Chockchaisawasdee et al. 2005; Nguyen et al. 2006; Nakkharat & Haltrich, 2006; Splechtna et al. 2007; Goulas et al. 2009; Neri et al. 2009; Maischberger et al. 2010; Iqbal et al. 2011; Liu et al. 2011; Rodriguez-Colinas et al. 2011; 2012; 2013; Nguyen et al. 2012; Osman et al. 2012; Urrutia et al. 2013; Wu et al. 2013; Yu & Sullivan, 2013; Arreola et al. 2014). *A. oryzae*, *Bacillus circulans*, *Cryptococcus laurentii*, *K. lactis*, and *Streptococcus thermophilus* are commercial sources of BGALs used for GOS production. When using these enzymes, yields of GOS formation ranging from 14 to 45 % are reported with 5 % initial lactose concentrations (Torres et al. 2010; Gosling et al. 2010). The use of rational design and enzyme engineering offers a means by which to improve the transglycosylation-to-hydrolysis (T/H) ratio to produce more useful enzyme variants giving higher GOS yields.

Alternatives to BGALs for these applications include many retaining *β*-glucosidases (BGL) that are capable of catalyzing transglycosylation as a side reaction to the functionally relevant hydrolytic cleavage of glycosidic bonds. Efforts to engineer BGLs for improved transglycosylation have been reported in the literature (Hansson et al. 2001; Feng et al. 2005; Wu et al. 2013), where the typical engineering strategy involves replacement of side chains in the aglycone (+1) and glycone (−1) subsites (Hansson et al. 2001; Feng et al. 2005). For engineering, it is advantageous to start with an enzyme that has evolved naturally to be functional under conditions that are relevant for the desired application, for instance high temperature. Thermophilic and hyperthermophilic bacteria are among the most useful microbial producers of highly stable and robust enzymes for bioprocesses. Among others, *Halothermothrix orenii* is a heterotrophic, halophilic, thermophilic, obligate anaerobic bacterium (Cayol et al. 1994) that produces a thermostable *β*-glucosidase, *Ho*BGLA (Mijts & Patel, 2001; Mavromatis et al. 2009; Kori et al. 2011; Hassan et al. 2015). We have reported previously the biochemical and structural characterization of *Ho*BGLA and shown that this GH1 glycosidase (www.cazy.org; Lombard et al. 2014) displays attractive properties relevant to GOS synthesis (Hassan et al. 2015). Specifically, the enzyme has *β*-galactosidase activity at high temperatures (65–70 °C) and within a broad pH range (4.5–7.5), conditions under which the wild-type enzyme also displays significant transgalactosylation activity to efficiently convert lactose into mainly *β-*d-Gal*p*-(1 → 3)-d-Lac (3′-galactosyl lactose; 3GALA) and *β-*d-Gal*p-*(1 → 6)-d-Lac (6′-galactosyl lactose; 6GALA).

Encouraged by the high natural transgalactosylation activity of *Ho*BGLA, and the general amenability of BGLs to be engineered toward improved T/H ratios, we set out in this study to investigate the possibility to further improve transgalactosylation activity and GOS yield for *Ho*BGLA by replacing amino acid side chains in, or near, the −1 subsite in positions previously shown to improve transglycosylation for related BGLs (Hansson et al. 2001; Feng et al. 2005; Lundemo et al. 2013; Teze et al. 2014). We present the production, biochemical characterization, and transgalactosylation analysis for six *Ho*BGLA active-site variants aimed at improving GOS production.

## Materials and methods

### Site-directed mutagenesis, expression, and purification of *Ho*BGLA variants

The cloning and expression of the *H. orenii bglA* gene (UniProtKB B8CYA8) have been reported previously (Hassan et al. 2015). The wild-type *bglA* gene cloned in the pNIC28-Bsa4 vector containing a cleavable N-terminal hexahistidine tag and the tobacco etch virus (TEV) protease cleavage site (sequence ^23^MHHHHHHSSGVDLGTENLYFQSM^−1^) (Savitsky et al. 2010) was used as template for site-directed mutagenesis to produce the single-replacement variants N222F, N294T, Y296F, N406I, F417Y, and F417S. Forward and reverse PCR primers were designed with the QuickChange® Primer Design Program from Agilent Technology. The forward primers are given in Table 1, and the reverse primers were the reverse complements of the forward primers. All PCR reactions, plasmid transformations, expression, cell harvest, and protein purification were performed as described for the *Ho*BGLA wild-type and active-site variants reported earlier (Hassan et al. 2015). Briefly, *Escherichia coli* BL21(DE3) cells were grown in Terrific Broth (TB) medium supplemented with 50 μg/mL kanamycin and glycerol as carbon source (60 mL per 600 mL), induced with 0.2 mM IPTG and cultivated at 18 °C for 16–18 h. Purification was performed as described previously (Hassan et al. 2015), using an initial step of Ni^2+^-charged immobilized metal affinity chromatography (IMAC), after which the His_6_ tag was removed using tobacco etch virus (TEV) protease. After tag removal, a second step of IMAC was performed (i.e*.*, reverse IMAC) where the TEV-treated target protein lacking the His_6_ tag was collected in the flow through and further purified using size exclusion chromatography on a HiLoad™ 16/60 Superdex™ 200 prep grade column (GE Healthcare Life Sciences) equilibrated with 20 mM HEPES (pH 7.0) and 150 mM NaCl.

**Table 1:** Forward PCR mutagenesis primers

N222F_fwd	5′-CGGGTAAGCAGGGGTTAAGAAGAGAGTAATACCAATCTCC-3′
N294T_fwd	5′-CCATCCTGGAGTAGTAAGTAATGCCCAGGAAGTCAA-3′
Y296F_fwd	5′-ACCACCATCCTGGAGAAGTAATTAATGCCCAGGAA-3′
N406I_fwd	5′-GCCATAGGCCCATTCAAAAATATCCATCAATGACCACAC-3′
F417Y_fwd	5′-CCTATGGCTATAGCAAGCGCTATGGTCTCATTTATG-3′
F417S_fwd	5′-TAATCAACATAAATGAGACCACTGCGCTTGCTATAGCCATAGGC-3′

### Hydrolytic activity assays of the *Ho*BGLA variants on cellobiose and lactose

The hydrolytic activity of *Ho*BGLA variants on cellobiose and lactose was assayed using the coupled glucose oxidase/peroxidase assay (Kunst et al. 1988) as described earlier for wild-type and mutant *Ho*BGLA (Hassan et al. 2015), the only differences being the buffer and temperature at which the assay was performed. The reactions were carried out at 70 °C in 20 mM HEPES buffer (pH 7.0) and 0.15 M NaCl with substrate concentrations in the range 2 to 120 mM for cellobiose, and 5 to 400 mM for lactose. Enzyme concentrations used were as follows: wild type, 0.085 mg/mL; N222F, 1.7 mg/mL; N294T, 4 mg/mL; Y296F, 5 mg/mL; F417S, 8.5 mg/mL; and F417Y, 4 mg/mL. One unit of lactose-hydrolyzing activity was defined as the amount of enzyme releasing 1 μmol of D-glucose per minute under the given conditions. One unit of cellobiose-hydrolyzing activity was defined as the amount of enzyme releasing 2 μmol of D-glucose per minute under similar conditions as described for determination of *β*-galactosidase activity using lactose as the substrate. Non-linear regression was used to derive the kinetic parameters, and the data were fitted to the Michaelis–Menten model using GraphPad Prism 6.0 for Mac (GraphPad Software, San Diego, CA, USA, www.graphpad.com). The apparent turnover values (*k*_cat,app_) were calculated using the experimentally determined *v*_max_ values and a molecular mass of 53 kDa for the enzyme.

### TLC screening for GOS production by *Ho*BGLA variants using cell lysates

To assess the transglycosylation activity of the *Ho*BGLA variants, synthesized GOS products were screened by thin-layer chromatography (TLC). To this end, the crude-cell extracts were incubated in the presence of 30 % lactose (*w*/*v*) at 70 °C and different durations, allowing denaturation of most endogenous *E. coli* proteins. Specifically, 2-mL overnight cultures of *E. coli* BL21(DE3) cells carrying the *p*NIC28-Bsa4-*HoBGLA* vectors were harvested by centrifugation, and the cell pellet resuspended in either 200 μL sodium phosphate buffer, pH 6.0, containing 300 g/L lactose, and 1 mM Mg^2+^ to increase enzyme stability (Nguyen et al. 2006; Iqbal et al. 2011; Hassan et al. 2015), or 200 μL sodium phosphate buffer, pH 6.0, containing 200 g/L cellobiose and 1 mM Mg^2+^. Cells were lysed by ultrasonication on ice. The resulting cell lysates were incubated at 70 °C with shaking (700 r.p.m.). In the case of lactose as substrate, the reaction was run for 3 and 4.5 h, after which samples were taken for analysis of GOS products. The samples were heated at 95 °C for 5 min and diluted 1:10, followed by loading of 1 μL sample on a TLC plate. In the case of cellobiose as substrate, the reaction was carried out for 2 h after which the enzymes were heat-inactivated for 5 min at 95 °C, and the carbohydrate content analyzed using TLC (2 μL of 1:10 dilution). HPTLC Li Chrosper®Silica gel 60 F_254_s (Merck) was used as adsorbent. Samples were applied on the plates and placed in the eluent (*n*-butanol–*n*-propanol–ethanol–water = 2:3:3:2). Visualization of the separated carbohydrates was performed by immersing the TLC plate in a staining solution (0.5 g thymol, 95 mL 96 % ethanol, 5 mL concentrated sulfuric acid) for 3 s and subsequent heating at 90 °C for approximately 1 min. Standards for lactose conversion included a mixture of glucose, galactose, and lactose (LGG); a purified GOS mixture with monosaccharides and lactose removed produced using *Lactobacillus* sp. *β*-galactosidase (Maischberger et al. 2008); and Vivinal®GOS (Borculo Domo, NL). For cellobiose conversion, cellobiose and glucose were used as standards.

For HPLC analysis of transglycosylation products obtained with purified enzymes, a volume of 50 μL enzyme (7 mg/mL) was mixed with 450 μL lactose (300 g/L) in 50 mM sodium phosphate buffer (pH 6) containing 1 mM MgCl_2_ and incubated with shaking at 70 °C at 600 r.p.m. for 12 h. Samples were withdrawn at different time points and heat-inactivated for 5 min at 95 °C, followed by HPLC analysis as described previously (Spechtna et al. 2007).

### Transgalactosylation of lactose using purified enzymes and analysis of galacto-oligosaccharides

A volume of 50 μL purified enzyme (7 mg/mL) of *Ho*BGLA variants was mixed with 450 μL lactose (300 g/L) in 50 mM sodium phosphate buffer (pH 6) containing 1 mM MgCl_2_ and incubated with shaking at 70 °C at 600 r.p.m. for 12 h. Samples were withdrawn at specific time intervals and immediately transferred to 99 °C for 5 min to inactivate the enzyme. Samples were stored at −18 °C for subsequent analysis.

The GOS mixtures were analyzed by high-performance anion exchange chromatography with pulsed amperometric detection (HPAEC-PAD). HPAEC-PAD analysis was carried out on a Dionex DX-500 system consisting of a GP50 gradient pump, an ED 40 electrochemical detector with a gold working electrode and an Ag/AgCl reference electrode, and Chromeleon version 6.5 (Dionex Corp., Sunnyvale, CA). All eluents were degassed by flushing with helium for 30 min. Separations were performed at room temperature on a CarboPac PA-1 column (4 mm × 250 mm) connected to a CarboPac PA-1 guard column (Dionex). Separation of D-glucose-, D-galactose, lactose, and allolactose was carried out with an isocratic run (45 min) with 15 mM NaOH at 1.0 mL/min, followed by 25-min elution with 100 mM NaOH (gradient 1). For separation of other GOSs, eluents A (100 mM NaOH) and B (100 mM NaOH and 150 mM NaAc) were mixed to form the following gradient: 98 % A from 0 to 10 min, 98 % A to 52 % A from 10 to 40 min, and then 52 % A for another 5 min (gradient 2). The column was washed with 20 % B for 10 min and re-equilibrated for 15 min with the starting conditions of the employed gradient.

Individual GOS components were identified by comparison to authentic material, specifically β*-*D-Gal*p*-(1 → 3)-D-Glc, β*-*D-Gal*p*-(1 → 6)-D-Glc, β*-*D-Gal*p-*(1 → 3)-D-Gal, β*-*D-Gal*p-*(1 → 4)-D-Gal, β*-*D-Gal*p-*(1 → 6)-D-Gal, β*-*D-Gal*p-*(1 → 3)-D-Lac, β*-*D-Gal*p-*(1 → 4)-D-Lac, and β*-*D-Gal*p*-(1 → 6)-D-Lac purchased from Carbosynth (Berkshire, UK). The degree of lactose conversion was calculated as percentage of lactose converted of initial lactose employed. The GOS yields were calculated as percentage of GOSs formed of total sugars.

## Results

### Construction and expression of *Ho*BGLA variants

Based on engineering studies on related GH1 *β*-glycosidases (Hansson et al. 2001; Feng et al. 2005; Wu et al. 2013), amino acids in the glycone (−1) and aglycone (+1) subsites of *Ho*BGLA were selected for mutagenesis with the aim to enhance the T/H ratio. The *β*-glucosidase *Tn*Bgl1A from *Thermotoga neapolitana* was engineered toward improved transglycosylation by replacing N^220^ in subsite +1 with phenylalanine. In *Tn*Bgl1A, this substitution caused significant improvement of the T/H ratio and higher yields of alkyl glycosides through transglycosylation (Lundemo et al. 2013). The corresponding residue in *Ho*BGLA is N^222^, and thus, the *Ho*BGLA variant N222F was considered an interesting candidate. Introducing a phenylalanine side chain would increase hydrophobicity of the +1 subsite and possibly increase the affinity for acceptors of less polar character than water, such as sugars, which theoretically may favor a higher T/H ratio.

In the case of *Thermus thermophilus β*-glycosidase Tt*β*-gly, directed-evolution experiments identified the replacements of N282T, N390I, and F401S in the vicinity of the −1 subsite as variants with improved T/H ratios (Feng et al. 2005; Teze et al. 2014). These residues are conserved in *Ho*BGLA and correspond to N^294^, N^406^, and F^417^. Moreover, the transgalactosylation activity increased by 22 % for the *Pyrococcus furiosus β*-glucosidase CelB variant F426Y (F^417^ in *Ho*BGLA) at low lactose concentrations compared with the wild type (Hansson et al. 2001). A comparison of the crystal structures of Tt*β*-gly N282T (PDB code 4BCE; Teze et al. 2014) and F401S (PDB code 3ZJK; Teze et al. 2014) with that of *Ho*BGLA (PDB code 4PTX; Hassan et al. 2015) suggested that these replacements may cause similar effects in *Ho*BGLA. Based on the above information, the *Ho*BGLA variants N294T, N406I, F417S, and F417Y were selected. Additional Tt*β*-gly variants with promising transglycosylation characteristics have been reported, such as R75A, W120C, N163A, and Y284F (Teze et al. 2014). These positions correspond to R^77^, W^122^, N^165^, and Y^296^ in *Ho*BGLA, respectively, and of these, the *Ho*BGLA Y296F replacement in subsite −1 was considered for further work.

**Fig. 1:**
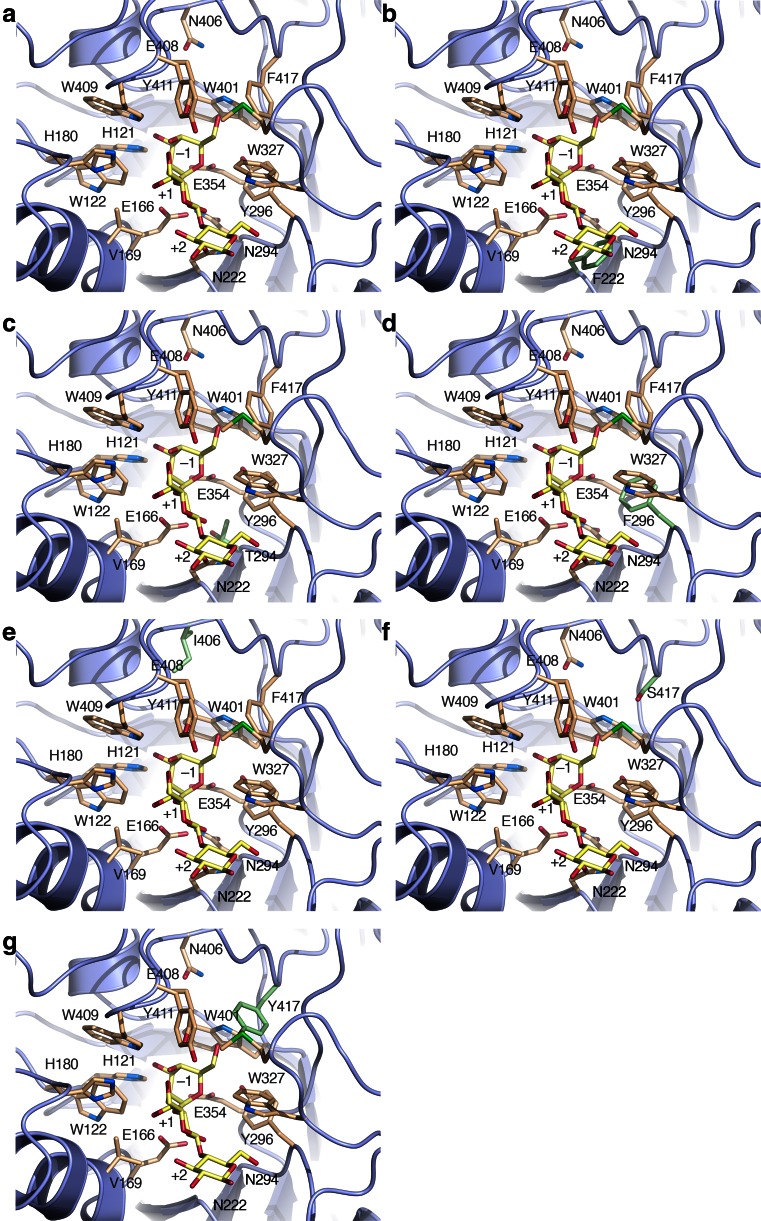
Structural details of mutation sites**. a** The active site of wild-type *Ho*BGLA with relevant residues shown (PDB code 4PTX; Hassan et al. 2015). The amino acid replacements were modeled in the crystal structure: **b** N222F, **c** N294T, **d** Y296F, **e** N406I, **f** F417Y, and **g** F417S. Mutated residues are highlighted in *green*. A previously modeled 3GALA molecule (Hassan et al. 2015) was shown to delineate the subsites −1, +1, and +2 and has been superimposed on the theoretical structural models of the variants (Color figure online)

In total, six *Ho*BGLA variants were designed rationally, including N222F, N294T, Y296F, N406I, F417Y, and F417S (Fig. 1), of which all but N406I could be expressed. The expression yields for the purified proteins (after reverse IMAC) were as follows: wild type, 7.5 mg/L culture (0.8 mg/g of wet cell mass); N222F, 1.0 mg/L culture (0.14 mg/g of wet cell mass); N294T, 2.9 mg/L culture (0.5 mg/g of wet cell mass); N406I, no expression; F417S, 3.8 mg/L culture (0.9 mg/g of wet cell mass); F417Y, 2.5 mg/L culture (0.4 mg/g of wet cell mass); and Y296F, 3.2 mg/L culture (0.5 mg/g of wet cell mass).

### Characterization of the hydrolytic activity of *Ho*BGLA mutants

The kinetic parameters (*v*_max_, *K*_m_, *k*_cat_/*K*_m_) for hydrolysis of cellobiose and lactose were determined for the *Ho*BGLA wild type and variants (Table 2). We previously reported the kinetics for the *Ho*BGLA-catalyzed hydrolysis of cellobiose and lactose at 50 °C (Hassan et al. 2015). The turnover number for cellobiose hydrolysis catalyzed by the wild type increases slightly (∼6 %) when raising the temperature to 70 °C, while a larger increase in apparent *k*_cat_ is observed for lactose hydrolysis (∼60 %). The changes in the *K*_m_ values in response to increasing temperature are more pronounced with *K*_m[cellobiose]_ decreasing 2.5-fold and *K*_m[lactose]_ dropping 6-fold. These changes are manifested as a 2.7-fold and 9-fold increase in specificity constant for cellobiose and lactose, respectively. This shows that wild-type *Ho*BGLA achieves improved specificity and catalytic efficiency for both cellobiose and lactose at 70 °C. Compared with the wild-type enzyme, all expressed variants show impaired hydrolytic activity for both substrates.

**Table 2:** Kinetic parameters for *β-*glucosidase and *β-*galactosidase activities

	d-cellobiose			d-lactose			Selectivity, cellobiose over lactose^b^
	*k*_cat,app_ (s^−1^)	*K*_m_ (mM)	*k*_cat,app_/*K*_m_ (mM^−1^ s^−1^)	*k*_cat,app_ (s^−1^)	*K*_m_ (mM)	*k*_cat,app_/*K*_m_ (s^−1^ mM^−1^)	(*k*_cat,app_/*K*_m[cel]_)/(*k*_cat,app_/*K*_m[lac]_)
WT^a^	366	25.4	14.4	231	154	1.54	9.4
WT	387 ± 15	10.0 ± 1.0	38.7	366 ± 17	26.3 ± 2.8	13.9	2.8
N222F	16.9 ± 1.3	93.2 ± 12.6	0.18	1.47 ± 0.05	41.3 ± 3.1	0.036	5.0
N294T	16.5 ± 0.4	9.0 ± 0.8	1.8	7.4 ± 0.2	30.8 ± 2.0	0.24	7.5
Y296F	8.5 ± 0.3	20.2 ± 1.6	0.42	6.9 ± 0.2	29.1 ± 2.3	0.24	1.8
F417Y	38.7 ± 0.6	3.5 ± 0.2	11.1	41.5 ± 1.2	10.3 ± 0.9	4.0	2.8
F417S	0.38 ± 0.01	11.9 ± 0.9	0.032	7.8 ± 0.4	23.2 ± 2.7	0.34	0.09

^a^Reaction performed at 50 °C; from Hassan et al. 2015; all other reactions performed at 70 °C

^b^A ratio >1 favors cellobiose hydrolysis, whereas a ratio <1 favors lactose hydrolysis

The variant N222F shows an ∼23-fold decrease in *k*_cat_, and 9-fold increase in *K*_m_ for cellobiose hydrolysis. The variant is also severely compromised catalytically when lactose is used as substrate (∼250-fold lower *k*_cat_ and ∼1.6-fold increase in *K*_m_). For cellobiose hydrolysis, the variant N294T shows an ∼23-fold decrease in *k*_cat_ (as for N222F), while *K*_m_ remains unchanged compared with the wild type. Using lactose as substrate, this mutant displays an almost 50-fold decrease in *k*_cat_ and only slightly higher *K*_m_ (17 %). The *Ho*BGLA variant Y296F hydrolyzes cellobiose ∼45 times slower than does the wild-type enzyme, and the affinity for cellobiose is reduced, as indicated by a twofold increase in *K*_m_ value. Turnover of lactose is affected slightly more (∼53-fold lower *k*_cat_), but with only minor impact on *K*_m_ (11 % increase).

The least affected *Ho*BGLA variant is F417Y, which, compared to the wild type, retains 10 and 11 % of the cellobiose and lactose turnover numbers, respectively. A concomitant drop in *K*_m_ and 3.5-fold decrease in specificity constant for both substrates accompany the decrease in apparent *k*_cat_. With cellobiose as substrate, the variant F417S shows the lowest turnover number (*k*_cat_ 0.38 s^−1^), which corresponds to a 1000-fold decrease in *k*_cat_, but an unperturbed *K*_m_ value. However, hydrolysis of lactose by this variant is less affected with a 47-fold decrease in turnover number (*k*_cat_ 7.8 s^−1^) and similar *K*_m_ compared with the wild type. Thus, the F417S replacement in *Ho*BGLA is detrimental for cellobiose hydrolysis whereas this variant remains relatively competent with respect to lactose hydrolysis.

### TLC screening for GOS production by *Ho*BGLA variants using cell lysates

**Fig. 2:**
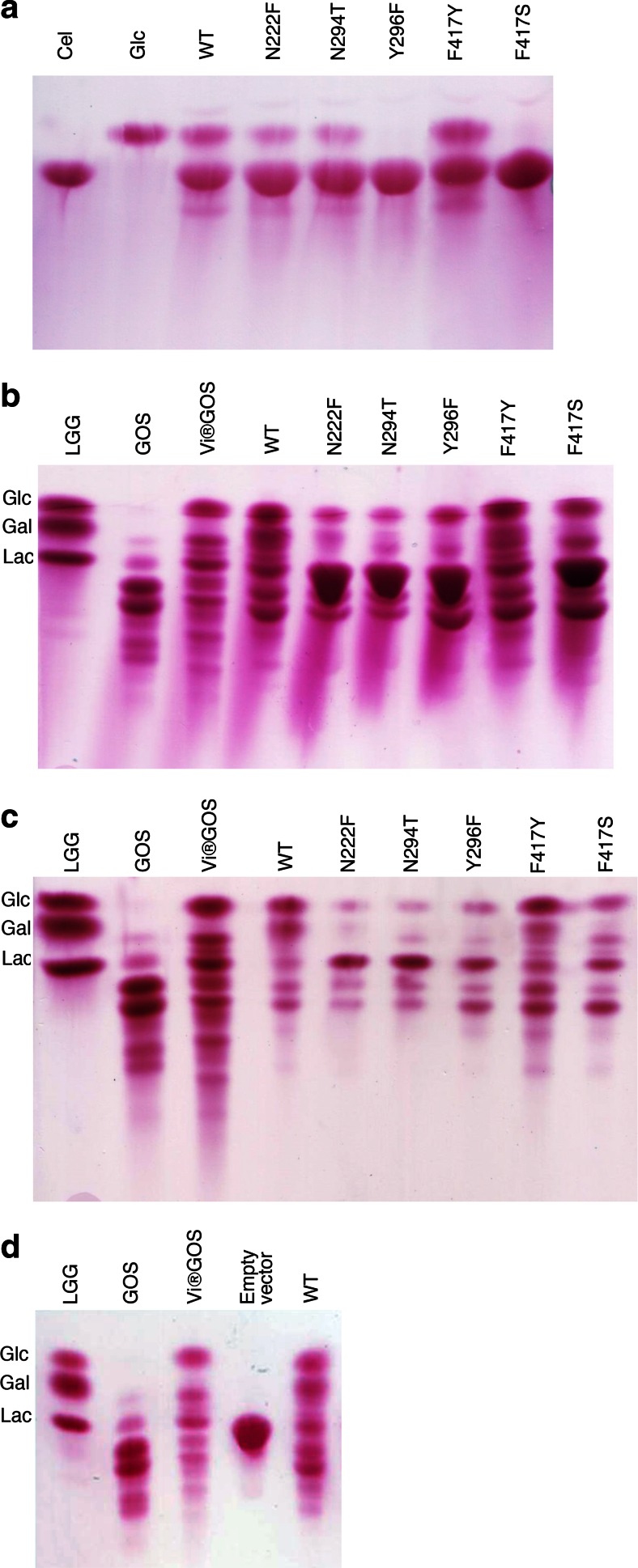
Cellobiose and lactose hydrolysis and transglycosylation using cell lysates**.** TLC analysis of *Ho*BGLA-catalyzed hydrolysis and transglycosylation in cell lysates of **a** cellobiose after 2 h, **b** lactose after 3 h, **c** lactose after 4.5 h; and **d** lactose after 4.5 h of reaction using cells carrying the expression vector without *Ho*BGLA insert as control. Standards used: LGG, lactose (Lac); galactose (Gal); glucose (Glc); GOS, purified GOS with monosaccharides and lactose removed; Vivinal®GOS; Cel, cellobiose

We have reported previously that wild-type *Ho*BGLA is able to transform lactose into GOSs efficiently and in high yields (Hassan et al. 2015). As an initial evaluation of the capacity of the *Ho*BGLA variants to synthesize GOSs, the crude lysates containing the respective variants were screened for their ability to hydrolyze cellobiose, and to convert lactose to products other than the hydrolysis products glucose and galactose using TLC (Fig. 2). As expected from the steady state kinetics (Table 2), all variants show hydrolytic activity on both cellobiose and lactose. The product patterns for cellobiose hydrolysis are similar for wild type, N222F, N294T, and F417Y (Fig. 2a). The absence of hydrolysis products from cellobiose for Y296F and F417S (Fig. 2a) under the conditions considered and the low associated turnover numbers, especially for F417S (Table 2), are consistent with poor performance of these mutants with cellobiose as substrate.

The variant F417Y displays product patterns from lactose hydrolysis similar to those of the wild type, whereas N222F, N294F, Y296F, and F417S show different patterns of GOSs formed (Fig. 2b, c). The variants Y296F, F417Y, and F417S seem to generate especially one GOS product in higher yields (i.e., the lower GOS band in Fig. 2b, c). Of these, F417Y shows the weakest signal for residual lactose, which reflects the higher *k*_cat,lac_ value of this variant compared with Y296F and F417S. As mentioned above, F417S shows a change in substrate selectivity from cellobiose to lactose, which could be useful for GOS production from substrate sources containing a mixture of cellobiose and lactose.

### Analysis of transgalactosylation activity and GOS production by *Ho*BGLA variants

The transgalactosylation activity of the *HoBGLA variants* was subsequently investigated in more detail using purified enzyme preparations. The reactions were performed at 70 °C with an initial lactose concentration of 300 g/L using the same amount of purified enzyme (∼0.35 mg). Three variants, Y296F, F417S, and F417Y, show improved total GOS yields compared with the wild-type enzyme (Table 3). The highest GOS yield of ∼57 % was obtained with F417Y, and F417Y also shows highest lactose conversion after 8 h among the variants tested. N294T is the least efficient in converting lactose and forming GOSs compared with the other variants and the wild-type enzyme under the conditions applied here.

**Table 3:** Degree of lactose conversion, GOS yield, and individual GOS components

Variants	WT	N222F	Y296F	F417S	N294T	F417Y
Degree of lactose conversion (%)	99.2	49.9	70.4	79.5	39.3	97.4
GOS yield (% mass of total sugars)	39.3	33.9	52.3	52.5	29.4	57.4
*GOS components* (% *mass of total GOS*)						
D-Gal*p*-(1 → 3)-d-Gal	8.2	0.0	0.0	0.0	0.0	2.8
D-Gal*p*-(1 → 6)-d-Gal	7.0	2.6	0.0	0.0	0.0	4.7
D-Gal*p*-(1 → 3)-d-Glc	8.8	3.7	10.5	25.1	22.8	13.6
D-Gal*p*-(1 → 6)-d-Glc	16.7	24.8	0.0	0.0	0.0	8.8
D-Gal*p*-(1 → 3)-d-Lac	5.1	10.9	16.9	6.2	31.0	13.4
D-Gal*p*-(1 → 4)-d-Lac	2.9	0.0	1.4	2.0	3.3	3.3
D-Gal*p*-(1 → 6)-d-Lac	45.4	57.9	66.3	66.5	42.6	53.1

Degree of lactose conversion, GOS yield, and individual GOS components produced by the transgalactosylation reaction of wild-type *Ho*BGLA and the variants using lactose as substrate after 8 h of reaction. The reactions were performed at 70 °C with an initial lactose concentration of 300 g/L in sodium phosphate buffer (pH 6.0) and 1 mM MgCl_2_ using 0.35 mg of purified enzyme

**Fig. 3:**
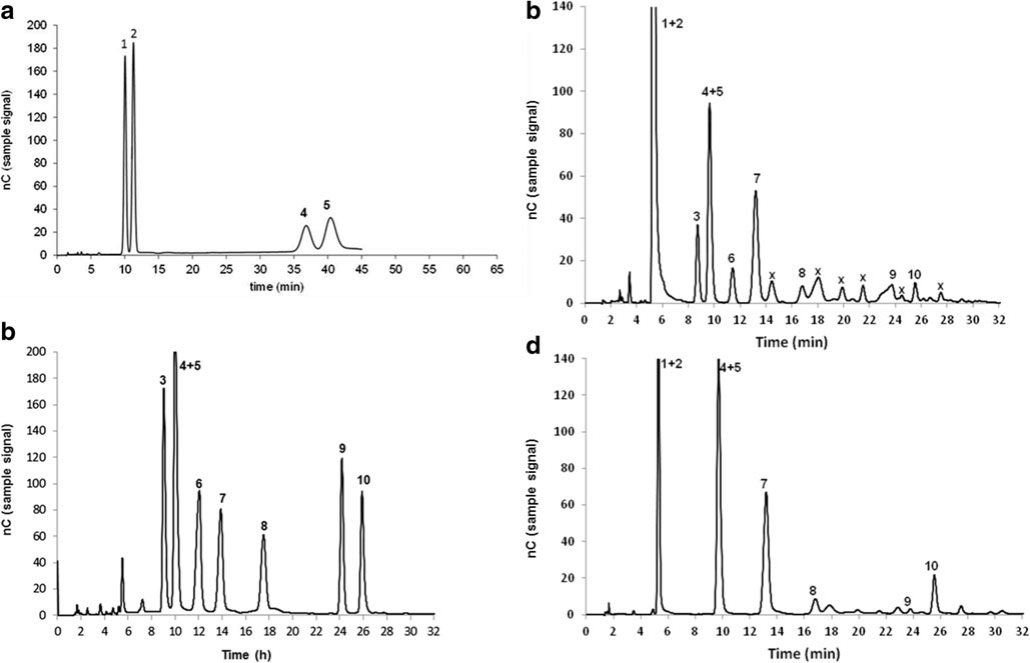
Separation and quantification by HPAEC-PAD. Separation and quantification by HPAEC-PAD of authentic standards (**a**, **b**) and of the GOS mixtures produced during lactose conversion catalyzed by wild-type *Ho*BGLA (**c**) and *Ho*BGLA F417S (**d**). The identified compounds are (*1*) galactose, (*2*) glucose, (*3*) D-Galp-(1 → 6)-D-Gal, (*4*) D-Galp-(1 → 6)-D-Glc (allolactose), (*5*) D-Galp-(1 → 4)-D-Glc (lactose), (*6*) D-Galp-(1 → 3)-D-Gal, (*7*) D-Galp-(1 → 6)-Lac, (*8*) D-Galp-(1 → 3)-D-Glc, (*9*) D-Galp-(1 → 4)-Lac, and (*10*) D-Galp-(1 → 3)-Lac. Products marked with an “*x*” were not identified. Different conditions were used for HPAEC-PAD analysis to separate D-glucose-, D-galactose, lactose, and allolactose (gradient 1, panel (**a**)) as well as the other oligosaccharides (gradient 2, panels (**b–d**)). Details are given in the “Materials and methods” section

All variants yield *β*-d-Gal*p*-(1 → 6)-Lac as the predominant oligosaccharide product (Table 3), at levels ranging from 43 to 67 % mass of the total GOSs produced. Y296F and F417S show almost identical GOS yields as well as the highest relative amounts of *β*-d-Gal*p*-(1 → 6)-Lac. Differences between these two variants during lactose transformation are that F417S converts lactose faster and that F417S forms the disaccharide *β*-d-Gal*p*-(1 → 3)-Glc as its second most frequent GOS product, whereas this is *β*-d-Gal*p*-(1 → 3)-Lac in the case of Y296F. Only four main GOS components were found in detectable amounts in the GOS mixtures formed when using Y296F and F417S, which were *β*-d-Gal*p*-(1 → 6)-Lac, *β*-d-Gal*p*-(1 → 3)-Glc, *β*-d-Gal*p*-(1 → 3)-Lac, and *β*-d-Gal*p*-(1 → 4)-Lac. Interestingly, F417S shows the same relative composition of main GOS components (calculated as percentage mass of the total GOSs) throughout the conversion (Table 4). Separation and quantification by HPAEC-PAD of individual GOS produced during lactose conversion catalyzed by wild-type *Ho*BGLA and F417S are given in Fig. 3.

**Table 4:** Time course of lactose conversion and formation of GOS by F417S

Time (h)	1	2	3	6	8
Degree of lactose conversion (%)	30.2	46.2	55.8	73.3	79.5
GOS yield (% mass of total sugars)	23.7	36.7	43.3	55.6	52.5
*GOS components* (% *mass of total GOS*)					
d-Gal*p*-(1 → 3)-d-Glc	18.8	23.1	24.7	25.5	25.1
d-Gal*p*-(1 → 4)-d-Lac	2.7	2.6	2.8	3.0	2.0
d-Gal*p*-(1 → 3)-d-Lac	7.4	6.9	6.5	6.1	6.2
d-Gal*p*-(1 → 6)-d-Lac	70.9	67.3	65.8	65.3	66.5

Time course of lactose conversion and formation of GOS during lactose conversion by the variant F417S. The reactions were performed at 70 °C at an initial lactose concentration of 300 g/L in sodium phosphate buffer (pH 6.0) and 1 mM MgCl_2_ using 0.35 mg of purified enzyme

## Discussion

When selecting the *Ho*BGLA variants to be engineered, the designs (Fig. 1) were guided by work performed on homologous GH1 enzymes. Compared with *Ho*BGLA N222F (Fig. 1b), the *Tn*Bgl1A variant N220F showed less dramatic effects on the kinetic parameters, i.e., *k*_cat_ and *K*_m_ increased by 4-fold and 2.8-fold, respectively, using *p*-nitrophenyl *β*-d-glucopyranoside (*p*NP-Glc) relative to the wild type (Lundemo et al. 2013). There is no crystal structure available for *Tn*Bgl1A, but molecular modeling suggests that the active site in *Tn*Bgl1A is sufficiently different compared with that of *Ho*BGLA to account for the discrepancy. In addition, different substrates were used, which makes a direct comparison difficult. That aside, the impact of the mutation appears to have a more drastic effect on *Ho*BGLA.

For the Tt*β*-gly N282T variant (corresponding to *Ho*BGLA N294T; Fig. 1c), *p*NP-Glc was hydrolyzed with 4-fold lower *k*_cat_ and 27-fold higher *K*_m_ (Feng et al. 2005), while hydrolysis of *p*NP-Gal was associated with a 8-fold lower *k*_cat_ and 6.5-fold higher *K*_m_ value compared with the wild-type enzyme. The crystal structure of Tt*β*-gly N282T (PDB code 4BCE) did not reveal any significant structural changes that could explain the altered performance for this variant (Teze et al. 2014). The discrepancy in catalytic performance between the *Ho*BGLA and Tt*β*-gly variants is difficult to rationalize from a structural viewpoint since the active sites are nearly identical, and the differences may be mainly accounted for by the different substrates used. Hydrolysis was severely compromised in the Tt*β*-gly variant Y284F (Teze et al. 2014) and *Agrobacterium β*-glycosidase Abg Y298F (Gebler et al. 1995), which correspond to *Ho*BGLA Y296F (Fig. 1d), and the tyrosine residue was assigned an important role to fine-tune the position of the nucleophile and to stabilize its deprotonated state (Gebler et al. 1995).

F417S (Fig. 1f) is the most catalytically impaired *Ho*BGLA variant with respect to the hydrolytic reaction, but with lactose performing considerably better than cellobiose. Similarly, the corresponding Tt*β*-gly variant F401S is also severely crippled catalytically, with no detectable hydrolytic activity on *p*-nitrophenyl *β*-d-glucoside (*p*NP-Glc) and *p*-nitrophenyl *β*-d-galactoside (*p*NP-Gal) (Feng et al. 2005). As for N282T, the crystal structure of Tt*β*-gly F401S (PDB code 3ZJK) offered no explanation for the loss of hydrolytic activity or improved T/H ratio for the mutants (Teze et al. 2014), but the phenylalanine residue was suggested to be selectively important for transition state (TS^*^) stabilization during the hydrolysis reaction, but of less significance for the transglycosylation reaction (Teze et al. 2014).

The rate of catalysis for cellobiose and lactose hydrolysis is only marginally affected in *Ho*BGLA F417Y (Fig. 1g), and decreased *K*_m_ values are observed for both substrates. For the corresponding *P. furiosus* CelB variant F426Y, the turnover number decreased by approximately 30 % while *K*_m_ was essentially unchanged using *p*NP-Glc (Hansson et al. 2001). To improve the yield of transglycosylation products for *P. furiosus* CelB variant F426Y, the complementary replacement M424K was made, generating the variant M424K/F426Y (Hansson et al. 2001). As for F426Y, the double mutant displayed somewhat lower turnover number while *K*_m_ increased 1.5-fold for *p*NP-Glc compared with the wild type. The position in *Ho*BGLA equivalent to M^424^ in CelB is K^415^, and *Ho*BGLA F417Y should therefore be compared with CelB M424K/F426Y.

By dividing the specificity constant for cellobiose hydrolysis by that of lactose hydrolysis, a selectivity ratio [(*k*_cat,app_/*K*_m[cellobiose]_)/(*k*_cat,app_/*K*_m[lactose]_)] is obtained, where a smaller ratio means improved performance with lactose over cellobiose as substrate. The selectivity ratio for the wild-type reaction with cellobiose measured at 50 and 70 °C shows that the preference for lactose is improved at higher temperatures, i.e., at 50 °C, cellobiose is ∼9 times favored over lactose, but at 70 °C, cellobiose is only 2.8 times more favored over lactose. Thus, in a reaction where both cellobiose and lactose are present, elevated temperature improves lactose hydrolysis relative to cellobiose hydrolysis. Interestingly, *Ho*BGLA F417S displays a change of substrate selectivity with lactose being favored 11-fold over cellobiose (selectivity ratio 0.09). Improved selectivity ratios for lactose are also observed for the other variants, although cellobiose remains the preferred substrate.

In this study, the objective was to evaluate whether *Ho*BGLA can be engineered toward improved transgalactosylation. With respect to transglycosylation performance, the variants F417S and F417Y are of particular interest to address. The *Ho*BGLA variant F417S (Table 3) produces mainly the trisaccharides *β*-d-Gal*p*-(1 → 6)-Lac and *β*-d-Gal*p*-(1 → 3)-Lac, and the disaccharides *β*-d-Gal*p*-(1 → 3)-Gal and *β*-d-Gal*p*-(1 → 3)-Glc, whereas for wild-type *Ho*BGLA, several other disaccharides are also produced. The percentages of disaccharides versus trisaccharides were 33.2 versus 66.5 % for F417Y, and 27.1 versus 72.7 % for F417S. The degree of lactose conversion of *Ho*BGLA F417Y is similar to that of the wild type, while the total GOSs has increased. The yield of total GOSs for F417Y is also close to that observed for some of the most efficient transglycosylating BGALs, e.g., recombinant *β*-galactosidase from *Bifidobacterium infantis* for which 63 % GOSs of the total sugars in the reaction mixture has been reported (Hung et al. 2001).

Several of the *Ho*BGLA variants reported here show interesting properties for lactose conversion and GOS production. The variant F417S, as well as Y296F, produces *β*-d-Gal*p*-(1 → 6)-Lac in significantly higher amounts than the wild type and the other variants. Amino acid replacements in the −1 subsite, such as in F417S or Y296F, resulted in a significantly different spectrum of GOS components formed and could be of interest for tailoring GOS mixtures, e.g*.*, for a higher content in trisaccharides. Variant F417Y is almost as efficient as wild-type *Ho*BGLA in converting lactose but more competent for overall GOS production. This work shows that the thermostable BGLA from *H. orenii* can be successfully engineered toward higher GOS production and different GOS distributions depending on the requirements. Although the present enzyme variants would not contribute to industrial GOS production, they offer molecular insights into improved GOS yields by *Ho*BGLA.
